# Bone-targeting engineered milk-derived extracellular vesicles for MRI-assisted therapy of osteoporosis

**DOI:** 10.1093/rb/rbae112

**Published:** 2024-09-13

**Authors:** Qing Huang, Yang Jiang, Yang Cao, Yunchuan Ding, Jinghui Cai, Tingqian Yang, Xin Zhou, Qiang Wu, Danyang Li, Qingyu Liu, Fangping Li

**Affiliations:** Department of Endocrinology, The Seventh Affiliated Hospital, Sun Yat-sen University, Shenzhen 518107, China; Research Center, The Seventh Affiliated Hospital, Sun Yat-sen University, Shenzhen 518107, China; Research Center, The Seventh Affiliated Hospital, Sun Yat-sen University, Shenzhen 518107, China; Department of Radiology, The Seventh Affiliated Hospital, Sun Yat-sen University, Shenzhen 518107, China; Department of Endocrinology, The Seventh Affiliated Hospital, Sun Yat-sen University, Shenzhen 518107, China; Department of Endocrinology, The Seventh Affiliated Hospital, Sun Yat-sen University, Shenzhen 518107, China; Department of Radiology, The Seventh Affiliated Hospital, Sun Yat-sen University, Shenzhen 518107, China; Department of Radiology, The Seventh Affiliated Hospital, Sun Yat-sen University, Shenzhen 518107, China; Research Center, The Seventh Affiliated Hospital, Sun Yat-sen University, Shenzhen 518107, China; Research Center, The Seventh Affiliated Hospital, Sun Yat-sen University, Shenzhen 518107, China; Research Center, The Seventh Affiliated Hospital, Sun Yat-sen University, Shenzhen 518107, China; Department of Radiology, The Seventh Affiliated Hospital, Sun Yat-sen University, Shenzhen 518107, China; Department of Endocrinology, The Seventh Affiliated Hospital, Sun Yat-sen University, Shenzhen 518107, China

**Keywords:** osteoporosis, milk-derived extracellular vesicles, SRT2104, bone-targeting peptide, MRI

## Abstract

The imbalance between osteoblasts and osteoclasts is the cause of osteoporosis. Milk-derived extracellular vesicles (mEVs), excellent drug delivery nanocarriers, can promote bone formation and inhibit bone resorption. In this study, we conjugated bone-targeting peptide (AspSerSer, DSS)_6_ to mEVs by click chemistry and then loaded with SRT2104, a SIRT1 (silent mating-type information regulation 2 homolog 1) agonist that was proofed to help reduce bone loss. The engineered (DSS)_6_-mEV-SRT2104 had the intrinsic anti-osteoporosis function of mEVs and SRT2104 to reverse the imbalance in bone homeostasis by simultaneously regulating osteogenesis and osteoclastogenesis. Furthermore, we labelled mEVs with MnB nanoparticles that can be used for the *in vivo* magnetic resonance imaging (MRI) visualization. The obtained nanocomposites significantly prevented bone loss in osteoporosis mice and increased bone mineral density, exhibiting superior bone accumulation under MRI. We believe the proposed (DSS)_6_-mEV-SRT2104/MnB provides a novel paradigm for osteoporosis treatment and monitoring.

## Graphical abstract

**Figure:**
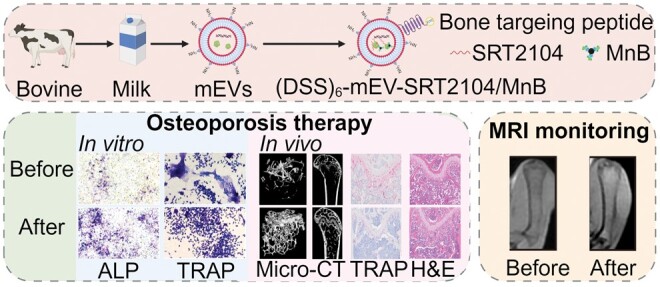

## Introduction

Osteoporosis, a systemic skeletal disease, is characterized by a reduction in bone mass, coupled with a profound deterioration in bone tissue microstructure, resulting in a significant increase in skeletal fragility and fracture risk [1]. During menopause, the acute loss of estrogen accelerates a rapid bone remodeling process, in which osteoclasts resorb more bone than osteoblasts form, resulting in a net bone loss [2]. The current clinically used drugs for osteoporosis treatment target either osteoblastic or osteoclastic activities, such as bisphosphonates [3], calcitonin [4] and denosumab [5]. However, these therapeutics have some limitations, including increased risk of cardiovascular adverse reactions, bone tumors and immune dysfunction [6]. Therefore, novel therapeutic strategies that can effectively re-establish the osteoblast/osteoclast balance and target osteoporotic bone lesions while minimizing systemic side effects are urgently needed.

Recent preclinical studies have reported a series of novel molecules that regulate osteoblast/osteoclast balance, aiming to increase bone density/mass [7]. For instance, silent mating-type information regulation 2 homolog 1 (SIRT1) was proven to promote new bone formation by the Wnt-β-catenin pathway [8–10], adipogenesis-related factors [11, 12], inhibition of extracellular matrix degradation [13, 14] and suppress osteoclastogenesis by regulating the NF-κB pathway [15, 16] and oxidative stress [17]. Hence, SIRT1 agonist, such as SRT2104, can help reduce bone loss and maintain the balance of bone metabolism for osteoporosis treatment, showing encouraging outcomes in enhancing bone health. It was reported that SRT2104 administrated orally to C57BL/6 mice significantly improved trabecular bone volume, trabecular connectivity and trabecular bone mineral density compared with control standard diet-fed animals [18]. However, this sacrifices its bioavailability to some extend. One solution to solve the above problem is to encapsulate SRT2104 into nanocarriers to obtain a new formulation that prolongs the blood circulation, increases bioavailability and reduces potential side effects.

Extracellular vesicles (EVs) are a heterogeneous collection of lipid bilayer-enclosed particles synthesized and secreted into the extracellular milieu by a wide variety of cell types [19]. They are stable in circulation, have low immunogenicity, able to carry functionally active biological molecules and be modified with target moieties. As a safe, economical and scalable alternative, milk-derived EVs (mEVs) have recently gained much popularity [20]. A number of recent studies have shown that mEVs have intrinsic therapeutic actions for various aspects such as intestinal health [21], regenerative properties in skin, hair and bone [22–24], anticancer activity [25] and immune modulation properties [26]. Specifically, it was reported that mEVs can accelerate osteoblastogenesis and reduce bone resorption [27, 28], improving the bone mineral density of glucocorticoid-induced osteoporosis mice [29]. However, targeted delivery to bone tissues remains challenging, limiting its further applications [30]. With abundant reactive surface chemistry, mEVs can be conveniently modified with targeting molecules, in which oligopeptide (AspSerSer)_6_, also termed as (DSS)_6_, with cell-penetrating ability could specifically target bone formation surface, showing great potential as an osteoblast targeting moiety [31, 32].

Theranostic nanoformulations have garnered significant attention due to their integration of diagnostic and therapeutic components within a single nanoscale platform [33]. Among different imaging modalities, magnetic resonance imaging (MRI) can provide non-invasive imaging and more detailed anatomic and functional information about the bone than X-ray-based modalities, making it an ideal method for continuous imaging of bone remodeling and the biodistribution of engineered EVs *in vivo* [34]. Conjugating MRI contrast agents with targeted EVs for molecular imaging is feasible. Mn-based contrast agents, one of the most commonly used MRI positive contrast agents, have been labelled with EVs and demonstrated promising performances, with no apparent biosafety concerns compared to Gd-based contrast agents [35, 36].

**Scheme 1.**
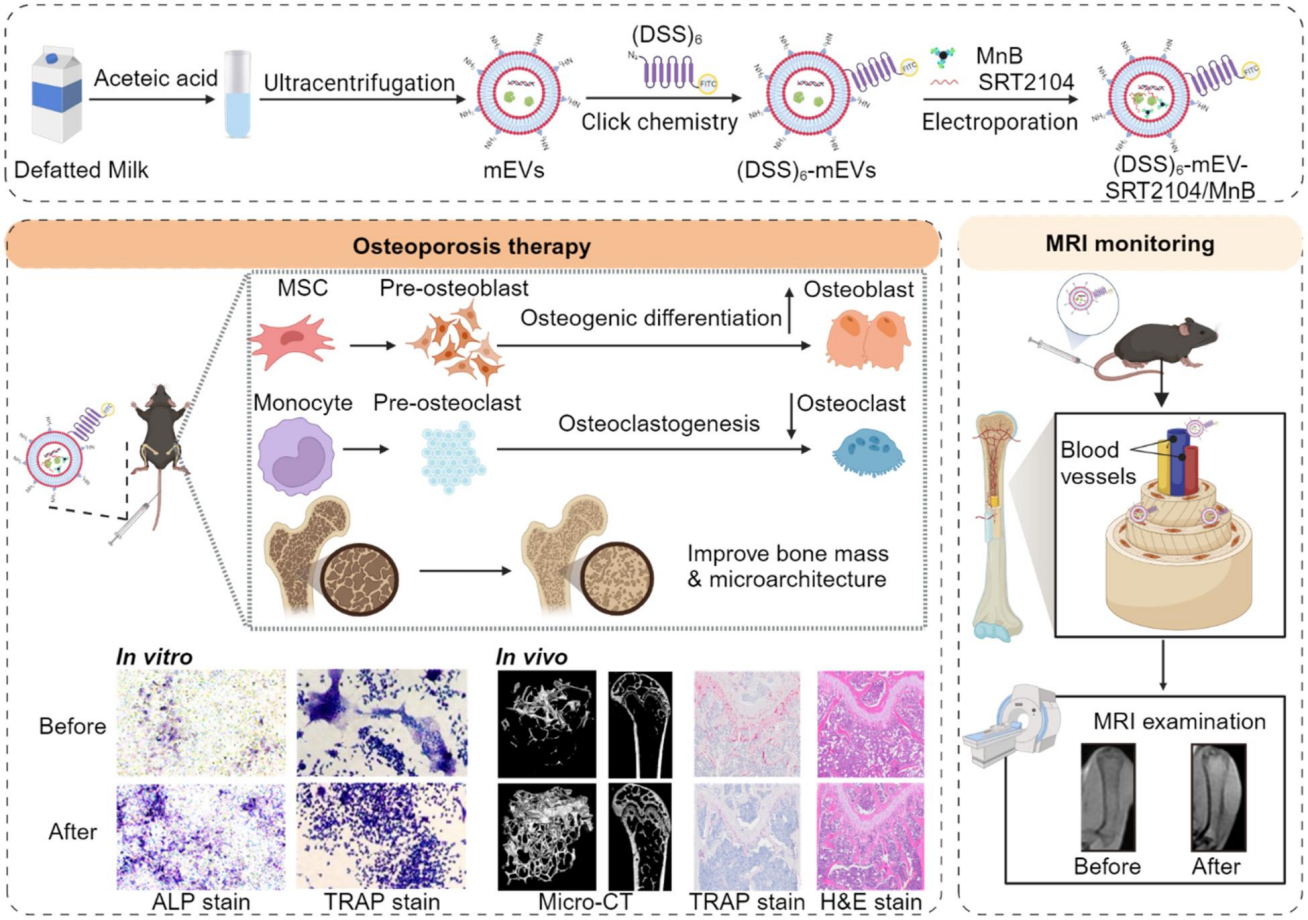
Schematic illustration of the development of bone-targeting engineered milk-derived extracellular vesicles for MRI-assisted therapy of osteoporosis.

In this study, we constructed an engineered (DSS)_6_-mEV-SRT2104/MnB for the treatment and monitoring of osteoporosis (Scheme 1). The (DSS)_6_ was anchored onto the mEVs membrane *via* click chemistry, and then SRT2104 and MnB nanoparticles (NPs) were loaded into mEVs *via* electroporation. We found mEV-SRT2104 could remarkably enhance osteogenic differentiation and inhibit excessive osteoclastogenesis *in vitro*. Additionally, we utilized an ovariectomized (OVX) mouse model to assess the therapeutic efficacy of the engineered mEVs assisted with MRI. We believe this research has developed a novel strategy for the treatment and monitoring of osteoporosis *via* engineered mEVs.

## Materials and methods

### Reagents

(DSS)_6_ (98% purity assayed by HPLC, with azide groups and FITC fluorescence, the sequence is FITC-Ahx-{Lys(N3)}DSSDSSDSSDSSDSSDSS) was purchased from IGE Biotechnology Ltd. SRT2104 (T6679, China) was purchased from TargetMol Chemicals Inc. Antibodies used in this study were rabbit polyclonal antibody against Alix (92800, Cell Signaling Technology), CD81 (27855, Proteintech), CD63 (25682, Proteintech), GAPDH (2118, Cell Signaling Technology), RUNX2 (20700, Proteintech), Calnexin (ab75801, Abcam), CTSK (ab187647, Abcam) and OPN (ab283656, Abcam).

### Isolation of mEVs

Preheat defatted milk for 10 min at 37°C, after which acetic acid (AA) was added (milk/AA = 100:1 (volume)). After stirring for 5 min, centrifuge the mixture at 13 000 *g* for 1 h at 4°C [37]. Next, filter the supernatant through a 0.22 µm membrane and obtain the whey. Then ultracentrifuge whey at 130 000 *g* for 90 min at 4°C by a SW32Ti rotor (Beckman Coulter). The mEVs pellets were resuspended and further ultracentrifuged. Finally, the mEVs pellets were resuspended and stored at −80°C. We examined the morphology of mEVs *via* a transmission electron microscope (TEM, Tokyo, Japan, JEM-1200EX). The size distribution and particle concentration were detected by a nanoflow cytometer (N30E Nanoflow Analyzer, Xiamen, China). The levels of protein markers of purified m-EVs (Alix, CD63 and CD81) and a negative marker (calnexin) were verified by western blotting. A bicinchoninic acid (BCA) protein assay kit was used to quantify the protein content of the mEVs. Following is the equation used to calculate the purity of mEVs:

Purity (particles/µg) = total number of mEVs/total protein mass of mEVs

### Modification of mEVs with bone-targeting peptide (DSS)_6_

Bone-targeting peptide was modified to mEVs *via* click chemistry. Firstly, DBCO-NHS easter was reacted with mEVs in a mass ratio of 3:20 for 4 h at room temperature to prepare DBCO-mEVs. The unreacted DBCO-NHS easter was removed through ultrafiltration with a molecular weight cut-off (MWCO) of 100 kDa. DBCO-mEVs was further reacted with bone-targeting peptide [the mass ratio of mEVs:(DSS)_6_ was 40:3] for 12 h at 4°C. After ultrafiltration, the free bone-targeting peptide was removed and the purified (DSS)_6_-mEVs were obtained [38]. Becton Dickinson’s flow cytometer was used to measure FITC fluorescence intensity.

### Synthesis and characterizations of MnB NPs

At room temperature, mix 2 ml of MnCl_2_ (40 mM) with an equal volume of bovine serum albumin (BSA) (40 mM). After adding 1 ml of 200 mM dimethylamine borane (DMAB), the reaction mixture was incubated for 1 h at 37°C. Next, 0.5 ml of 2 mg/ml solution of NaBH_4_ was added dropwise, and the mixture was stirred for 2 h. The purified MnB NPs were obtained after removing unreacted substances by dialysis. We measured the size and zeta potential of MnB NPs using dynamic light scattering (DLS) (Malvern Zetasizer Nano ZS, UK). The stability of MnB NPs was evaluated by measuring the size by DLS for 7 days in DI water. MnB NPs at a range of concentrations (0.1–0.4 mM) were prepared in distilled water. The longitudinal (*T*_1_) relaxation times of MnB NPs were measured with a 3.0 T clinical MRI scanner (SIGNA™ Architect, GE) using a custom-built volume coil with *T*_1_ mapping FARE sequence. The relaxation rate, *r*_1_, was determined by plotting 1/*T*_1_ versus MnB NP concentrations.

### Preparation of (DSS)_6_-mEV-SRT2104/MnB

About 25 μg of (DSS)_6_-mEVs, 7 μg of SRT2104 and 25 μg of MnB NPs were gently mixed with 500 μl PBS containing 5% alginate. An electroporation system (Bio-Rad) was used to electroporate the mixture in a 4 mm cuvette at 400 V and 150 μF and then incubated for 30 min at 37°C. After that, free MnB NPs were removed from the mixture by centrifuging for 1 h at 20 000 *g*. Finally, to remove free SRT2104, three washing steps with PBS using ultrafiltration (100 kDa MWCO, 10–20 min, 3000 g, 4°C) were performed. The purified (DSS)_6_-mEV-SRT2104/MnB was resuspended in PBS. The morphology of (DSS)_6_-mEV-SRT2104/MnB was characterized by TEM. The size and the concentration of (DSS)_6_-mEV-SRT2104/MnB were analysed by nanoparticle tracking analysis [NTA, (NanoSight Technology, Malvern, UK)]. The Mn^2+^ content within the mEVs was quantified by inductively coupled plasma mass spectroscopy (ICP-MS) (OpRIM 8300, PerkinElmer).

### Cell culture

MC3T3-E1 and RAW264.7 cells were cultured in alpha-MEM and DMEM containing 1% penicillin–streptomycin and 10% fetal bovine serum. A temperature of 37°C with 95% humidity and 5% CO_2_ was used for the incubation and fluid changes were performed for 2 days. The cells were digested when the cell density reached 80% of the culture dish's capacity and passaged.

### Alkaline phosphatase (ALP) staining

In six-well plates, MC3T3-E1 cells were seeded at a density of 1 × 10^5^ cells/well. About 10 mM β-glycerophosphate (Macklin, China) and 50 μg/ml ascorbic acid (Sigma, USA) were added to the medium after 24 h. mEV-SRT2104 was added and the medium was changed every 3 days. After 7 days, evaluate the production of ALP using a BCIP/NBT Alkaline Phosphatase Color Development Kit (Beyotime, China). Osteogenic induction medium (OIM), 25 μg/mL mEVs (protein content) and 1 μM SRT2104 were performed in the same condition.

### Tartrate-resistant acid phosphatase (TRAP) staining

In 96-well plates, RAW264.7 cells were reseeded at a density of 2 × 10^3^ cells/well. About 50 ng/ml RANKL (PeproTech, China) was added to the medium after 24 h. They were divided into RANKL group, 25 μg/ml mEVs (protein content) group, 1 μM SRT2104 group and mEV-SRT2104 group. Change the medium every 2 days. After 5 days, evaluate the production of TRAP by using a TRAP Stain Kit (Solarbio, China).

### Real-time qPCR

Extract total RNA after culturing MC3T3-E1 and RAW264.7 cells for 7 days. Sample concentration and purity were assessed using a NanoDrop-2000 spectrophotometer (Thermo). Then HiScript II Q RT SuperMix for qPCR (+gDNA wiper) (Vazyme, China) was used for reverse-transcription and SYBR Prime Script kit (Vazyme, China) for Real-time PCR. To normalize the outcome, the expression of GAPDH was chosen. Sequences of related genes were shown below:

mGAPDH, 5′-CGACTTCAACAGCAACTCCCACTCTTCC-3′ (sense)

5′-TGGGTGGTCCAGGGTTTCTTACTCCTT-3′ (antisense)

mRUNX2, 5′-AGAGTCAGATTACAGATCCCAGG-3′ (sense)

5′-TGGCTCTTCTTACTGAGAGAGG-3′ (antisense)

mALP, 5′-TGGACGGTGAACGGGAAAAT-3′ (sense)

5′-TAGTTCTGCTCATGGACGCC-3′ (antisense)

mOPN, 5′-GCTTGGCTTATGGACTGAGGTC-3′ (sense)

5′-CCTTAGACTCACCGCTCTTCATG-3′ (antisense)

mRANK, 5′-ATCATCTTCGGCGTTTAC-3′ (sense)

5′-CTTCTTGCTGACTGGAGG-3′ (antisense)

mCTSK, 5′-AGCAGAACGGAGGCATTGACTC-3′ (sense)

5′-CCCTCTGCATTTAGCTGCCTTTG-3′ (antisense)

mNFATC1, 5′-GGTGCCTTTTGCGAGCAGTATC-3′ (sense)

5′-CGTATGGACCAGAATGTGACGG-3′ (antisense)

### Western blot

The cells were cultured for 7 days. Proteins were separated using an SDS-PAGE gel and transferred to a polyvinylidene fluoride membrane. Primary antibodies against RUNX2 (1:1000), OPN (1:1000), CTSK (1:5000) and GAPDH (1:1000) were incubated at 4°C for 12 h. A secondary antibody against rabbit-HRP was used (Beyotime, China). With a chemiluminescence detection system (Bio-Rad, USA), protein bands were scanned and examined. The endogenous control employed was GAPDH.

### Cellular uptake of mEVs and (DSS)_6_-mEVs

Flow cytometry and an inverted fluorescent microscope were used to assess cellular uptake. The obtained mEVs and (DSS)_6_-mEVs were labelled in red using the Dil membrane dye (Macklin, China) at 37°C for 30 min. A centrifuge at 3000 *g* for 10 min was used to remove the free dye. In 6-well plates, MC3T3-E1 cells were seeded at a density of 1 × 10^4^ cells/well. Then change the medium for alpha-MEM containing 25 μg/ml mEVs or (DSS)_6_-mEVs labelled with Dil at 37°C for 4 h. For an inverted fluorescent microscope, 4% paraformaldehyde was applied to the cells for 15 min, followed by 10 min of DAPI staining. For flow cytometry, the cells were harvested and the Dil fluorescence intensity was analysed.

### MRI examination

The MRI examination of all samples was tested by a 3.0 T SIGNA™ Architect scanner (GE). The following parameters were established for the spin-echo sequence, which was used to obtain T1-weighted MRI images: 450.0 ms for the repetition time (TR) and 5.3 ms for the echo time (TE), field of view (FOV) of 80 × 80 mm^2^, flip angle (FA) of 90° and number of acquisition (NA) of 4. (DSS)_6_-mEVs-SRT2104/MnB and mEVs-SRT2104/MnB were prepared according to the method described above. Initially, 10 μg of each sample were incubated with 100 μg hydroxyapatite (HAp) suspensions and gently shaken for 2 h, and centrifugated for 10 min at 4000 rpm to separate the HAp and unbound mEVs. The supernatants were collected and imaged using a 3.0 T MRI scanner (SIGNA™ Architect, GE). Furthermore, the intensities of the supernatants were quantified using signal-to-noise ratio (SNR) measurements.

The biodistribution in normal 12-week-old C57BL/6 mice of three groups [free MnB, mEV-SRT2104/MnB and (DSS)_6_-mEV-SRT2104/MnB] was assessed at various time intervals using a clinical 3.0 T scanner (SIGNA™ Architect, GE) equipped with a mouse coil. During the MRI experiments, all mice were anesthetized with 2.0% isoflurane in oxygen, without assisted ventilation, enabling spontaneous breathing. Abdominal images were acquired using a T_1_-weighted spin-echo sequence (TR of 504 ms, TE of 5.01 ms, FOV of 30 × 30 mm^2^, FA of 90° and MTX of 200 × 200). Femur images were captured using a similar T_1_-weighted sequence with adjusted parameters (TR of 509 ms, TE of 20.0 ms, FOV of 80 × 80 mm^2^, FA of 100° and MTX of 256 × 256). Furthermore, the intensities of different organs were quantified using SNR measurements.

At the beginning of the therapy study (week 4), we randomly divided nine OVX mice into three groups, each receiving an intravenous injection of 0.1 ml of either mEV-MnB, mEV-SRT2104/MnB or (DSS)_6_-mEV-SRT2104/MnB (1 mg/ml protein), for subsequent MRI analysis. MRI examinations were repeated to assess the outcomes after therapy at week 10.

### Biodistribution of (DSS)_6_-mEV-SRT2104/MnB

To explore the *in vivo* distribution of (DSS)_6_-mEVs, we randomly divided 18 12-week-old female C57BL/6 mice into three groups [free-DiR, DiR-labelled mEV-SRT2104/MnB and DiR-labelled (DSS)_6_-mEVs]. About 150 μl of DiR-labelled mEVs or (DSS)_6_-mEVs (the protein concentration of mEVs: 1 mg/mL, DiR: 8 μg) was intravenously injected. At 4 and 24 h after injection, groups of three mice were euthanized. Using the *In Vivo* Imaging System (IVIS) with a 0.5 s exposure time (PerkinElmer, Hopkinton, MA, USA), the fluorescence intensity was assessed after the heart, liver, spleen, lung, kidney and long bones were isolated.

### Establishment of OVX mouse model

All animal experiments and procedures were approved by the Institutional Animal Care and Use Committee and Laboratory Animal Welfare and Ethics Committee at Shenzhen TopBiotech Co., Ltd (TOPGM-IACUC-2023-0084). To establish the OVX-induced osteoporotic mouse model, a midline incision in the dorsal skin and muscle layer exposed the bilateral ovaries of 12-week-old C57BL/6 female mice under anesthesia. The bilateral ovaries were removed following the ligation of the uterine horn. In the sham group, anesthesia, incisions, bilateral ovaries exposure and incision closure were performed without bilateral ovaries being removed [39]. The osteoporotic mouse model was successfully established four weeks after the surgery. We randomly divided the mice into the following seven groups: (*n *=* *5): sham, OVX, mEVs, SRT2104, mEV-SRT2104, (DSS)_6_-mEV-SRT2104 and zoledronic acid (ZOL) groups (Figure 4A). The amount of SRT2104 was estimated according to the previous study [18]. The dosage for each mouse *via* intravenous injection of SRT2104 and mEVs was 250 and 150 μg, respectively. The heart, liver, spleen, lung, kidney, femurs and serum were collected after 6 weeks of treatment.

### Micro-CT analysis

Femurs were scanned with a ZKKS micro-CT after being fixed for 24 h in 4% paraformaldehyde. The trabecular region that extends 240 pixels proximally, beginning 40 pixels below the growth plate, was chosen to gather the trabecular bone parameters. After that, 3D images were created by reconstructing the slices of the structure image. Bone volume fraction (BV/TV), trabecular number (Tb.N), trabecular thickness (Tb.Th), trabecular separation (Tb.Sp) and bone mineral density (BMD) were measured in the region of interest.

### Histological analysis

After a month of decalcification, femurs were dried out and embedded in paraffin. Sections (5 μm) were cut and stained using either TRAP or hematoxylin and eosin (H&E). Quantitative evaluation of bone trabeculae and osteoclast area was performed using ImageJ.

### Statistical analysis

We repeated all experiments at least three times, and the mean ± standard deviation was presented for the data. Two groups of data were compared using the paired *t*-test. When *P < 0.05, **P < 0.01, ***P < 0.001, the difference was considered significant.

## Results

### Preparation and characterization of (DSS)_6_-mEV-SRT2104/MnB

**Figure 1.**
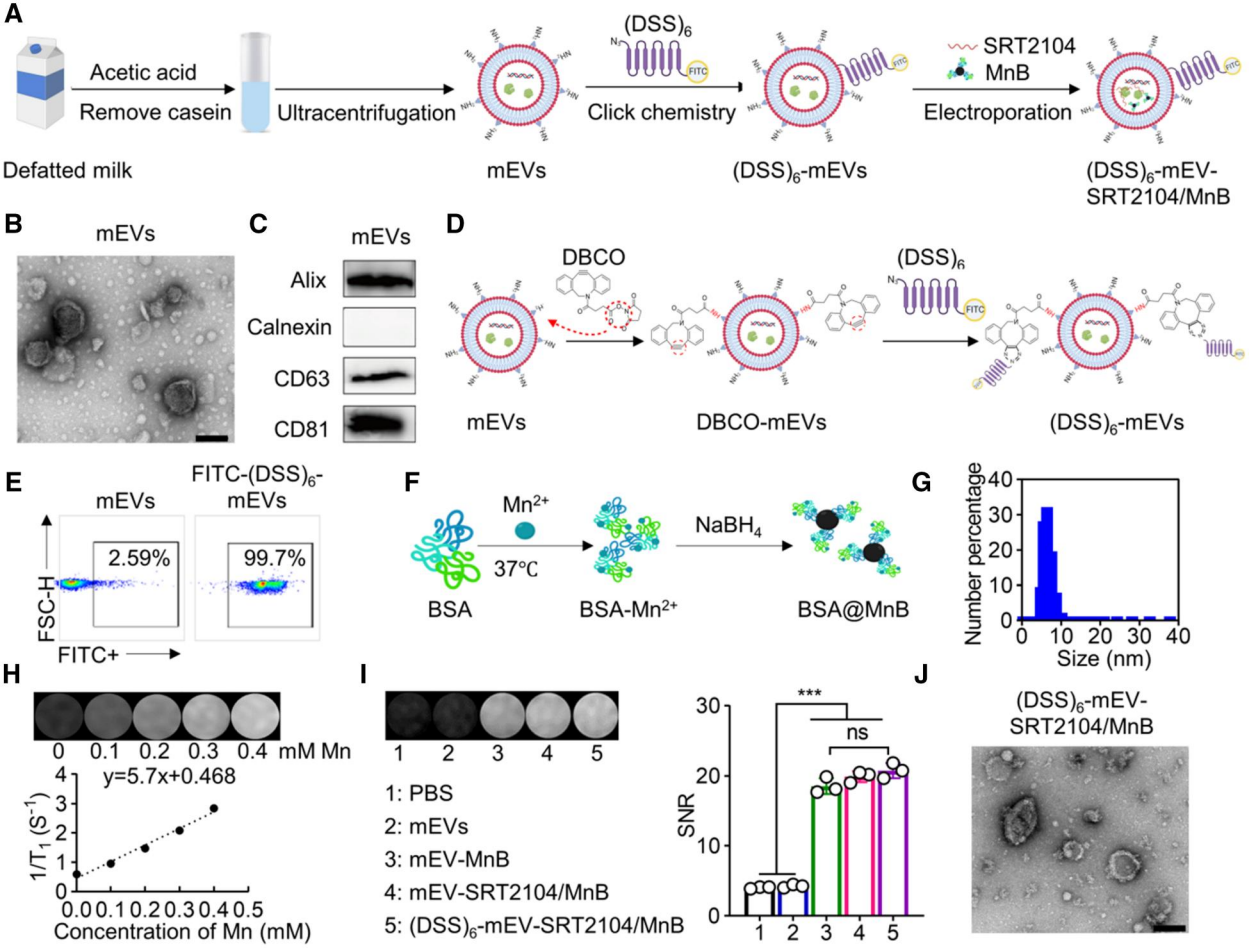
Preparation and characterization of (DSS)_6_-mEV-SRT2104/MnB. (**A**) Schematic illustration of the preparation procedure of (DSS)_6_-mEV-SRT2104/MnB. (**B**) TEM images of mEVs. Scale bar = 100 nm. (**C**) Protein marker characterization of mEVs *via* Western blot. (**D**) Schematic illustration of the preparation of (DSS)_6_-mEVs *via* click chemistry. (**E**) The characterization of (DSS)_6_-mEVs by flow cytometry. (**F**) Schematic illustration of the synthesis of MnB NPs. (**G**) Hydrodynamic size of MnB NPs *via* DLS. (**H**) T_1_-weighted MR images and 1/T_1_ of MnB NPs *via* 3.0 T clinical MRI scanner. (**I**) T_1_-weighted MR images of different samples and the corresponding SNR. (**J**) TEM images of (DSS)_6_-mEV-SRT2104/MnB. Scale bar = 100 nm (ns, no significant; ***P < 0.001).

mEVs were firstly purified from defatted bovine milk *via* acid precipitation and ultracentrifugation (Figure 1A). The morphology of the obtained mEVs was characterized by TEM, which displayed typical double-concave disc-shaped vesicular structures, indicating the successful preparation of mEVs (Figure 1B). Nanoflow results displayed that mEVs have a diameter of 66.5 ± 16.0 nm and their corresponding zeta potential was −15.2 ± 10.2 mV (Supplementary Figure S1A). The purity of mEVs was 2.0 × 10^9^ particles/µg protein (Supplementary Figure S1C), indicating a satisfactory purity of the mEVs for subsequent modifications and applications. The marker proteins of mEVs were characterized by western blotting, showing that the positive marker proteins Alix, CD63 and CD81 were expressed, while the negative marker protein calnexin was not detected (Figure 1C). They demonstrated the successful obtaining of mEVs.

Next, (DSS)_6_ was modified (Figure 1D) on mEVs *via* click chemistry [40]. Briefly, DBCO groups were introduced on the surface of mEVs by cross-linking mEVs with DBCO-NHS and reacting with azide-functionalized (DSS)_6_ to form stable triazole linkages using bio-orthogonal copper-free click chemistry. The flow cytometry results showed after modification, the FITC-(DSS)_6_-mEVs positive population was around 99.7% (Figure 1E), which indicated the successful modification of mEVs with (DSS)_6_.

To non-invasively visualize mEVs in clinical practice, MRI contrast agent MnB NPs were synthesized for the labelling of mEVs (Figure 1F). The diameter and the zeta potential of MnB NPs were 6.01 ± 1.32 nm and −17.32 ± 7.20 mV by DLS, respectively (Figure 1G). Hydrodynamic size results showed MnB NPs have high stability in DI water at 4°C for 7 days (Supplementary Figure S1B). The longitudinal relaxivity value of MnB NPs was calculated based on the plots of 1/*T*_1_ as a function of the Mn concentration on a 3.0 T clinical MRI scanner (SIGNA™ Architect, GE), yielding a value of 5.7 mM^−1^ s^−1^ (Figure 1H).

Finally, we incorporated MnB NPs and SRT2104 into (DSS)_6_-mEVs by electroporation. The size and the zeta potential of (DSS)_6_-mEV-SRT2104/MnB were 115.1 ± 50.5 nm and −20.7 ± 5.7 mV, respectively (Supplementary Figure S1D). We further confirmed that the loading of SRT2104 and MnB NPs into mEVs did not affect the imaging performance of MnB NPs (Figure 1I). The morphology of the obtained (DSS)_6_-mEV-SRT2104/MnB showed similar characteristics compared with mEVs by TEM (Figure 1J). And the loading efficiency of MnB is 15%. Collectively, the above results demonstrated that we have successfully constructed (DSS)_6_-mEV-SRT2104/MnB.

### mEV-SRT2104 promotes MC3T3-E1 oriented osteoblast differentiation and inhibits RAW264.7 oriented osteoclast differentiation

**Figure 2.**
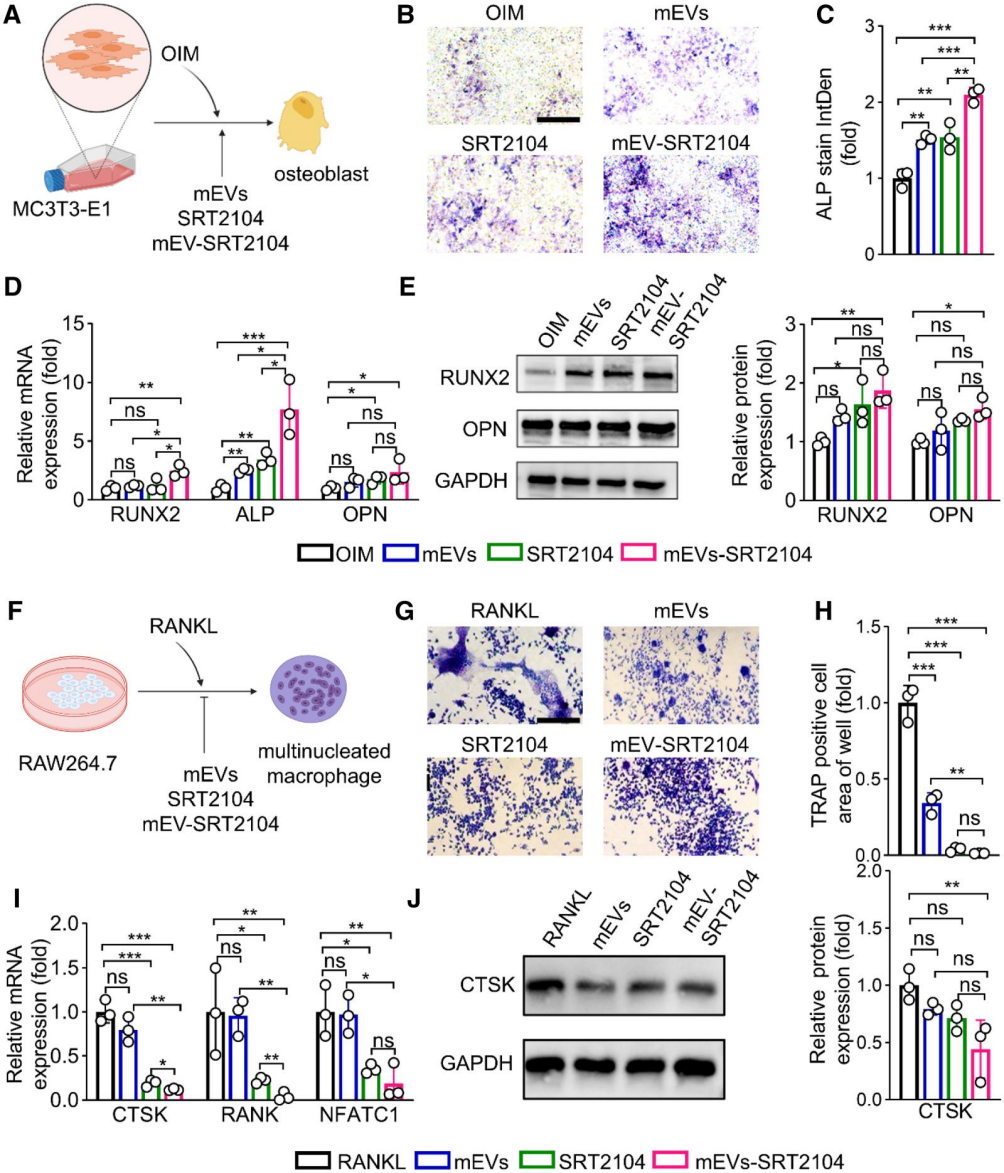
The effects of mEV-SRT2104 on osteoblasts and osteoclasts *in vitro*. (**A**) Schematic illustration of the osteogenic differentiation of MC3T3-E1 cells. (**B**) ALP staining of MC3T3-E1 cells after treatment for 7 days and (**C**) the quantification analysis of staining integrated density according to (**B**). Scale bar = 200 μm. (**D**) Relative mRNA expression of RUNX2, ALP and OPN after treatment for 7 days. (**E**) Western blot assay of protein RUNX2 and OPN after treatment for 7 days. (**F**) Schematic illustration of the osteoclast differentiation of RAW264.7 cells. (**G**) TRAP staining of RAW264.7 cells after treatment for 5 days and (**H**) the quantification analysis of TRAP-positive cell area. Scale bar = 200 μm. (**I**) Relative mRNA expression of CTSK, RANK and NFATC1. (**J**) Western blot assay of CTSK protein (ns, no significance; *P < 0.05, **P < 0.01, ***P < 0.001).

MC3T3-E1 cells were cultured with alpha-MEM media, then differentiated to osteoblasts with OIM (Figure 2A). According to the ALP staining, mEVs and SRT2104 caused a slight production of ALP in MC3T3-E1 while the mEV-SRT2104 group exhibited significantly greater efficiency in promoting the production of ALP than mEVs or SRT2104 (Figure 2B). Additional quantitative measurement showed that the mEVs and SRT2104 increased the expression of ALP by 1.5 folds compared to the OIM group. In comparison, the mEV-SRT2104 increased by 2.1 folds, indicating that mEV-SRT2104 demonstrated superior osteogenesis capabilities than mEVs or SRT2104 alone (Figure 2C). mRNAs and proteins associated with osteogenic differentiation, including ALP, osteopontin (OPN), RUNX2 were characterized *via* RT-qPCR and western blotting. In Figure 2D, following 7 days of culture, compared with OIM, the mEVs or SRT2104 treatment elevated osteoblast-specific gene mRNA levels modestly. Whereas the mEV-SRT2104 upregulated the mRNA levels of RUNX2 by 2.5 folds, ALP by 7.7 folds and OPN by 1.8 folds, respectively, demonstrating a more pronounced increase in osteoblast-specific gene expression. We performed western blotting studies further to assess the expressions of OPN and RUNX2 proteins. It was found that the levels of RUNX2 increased by 1.4, 1.6 and 1.9 folds in mEVs, SRT2104 and mEV-SRT2104, respectively. Similarly, the protein levels of OPN exhibited a 1.3-fold increase after mEV-SRT2104 treatment (Figure 2E). These results indicated that mEV-SRT2104 has shown superiority in promoting osteogenesis.

Next, we investigated the effects of mEV-SRT2104 on the osteoclast differentiation of RAW264.7 cells (Figure 2F). The results of TRAP staining showed that SRT2104 and mEV-SRT2104 groups had significantly fewer osteoclasts than other groups after osteoclastic differentiation for 5 days (Figure 2G and H). The osteoclast-specific mRNAs and proteins including CTSK, RANK and NFATC1 [41] were further detected. As displayed in Figure 2I, compared with the RANKL group, the SRT2104 and mEV-SRT2104 groups significantly downregulated the mRNA levels of CTSK by 5.1 folds and 8.4 folds, RANK by 4.5 folds and 24.2 folds and NFATC1 by 2.8 folds and 5.2 folds, respectively. Whereas, the mEVs group did not impact the expression of osteoclast-specific mRNAs after 7 days of culture, and the relative protein levels of CTSK decreased 1.3, 1.4 and 2.3 folds after mEVs, SRT2104 and mEV-SRT2104 treatment, respectively (Figure 2J). The results indicated that mEV-SRT2104 also significantly inhibited osteoclast.

### Bone-targeting capability *in vitro* and *vivo*

**Figure 3.**
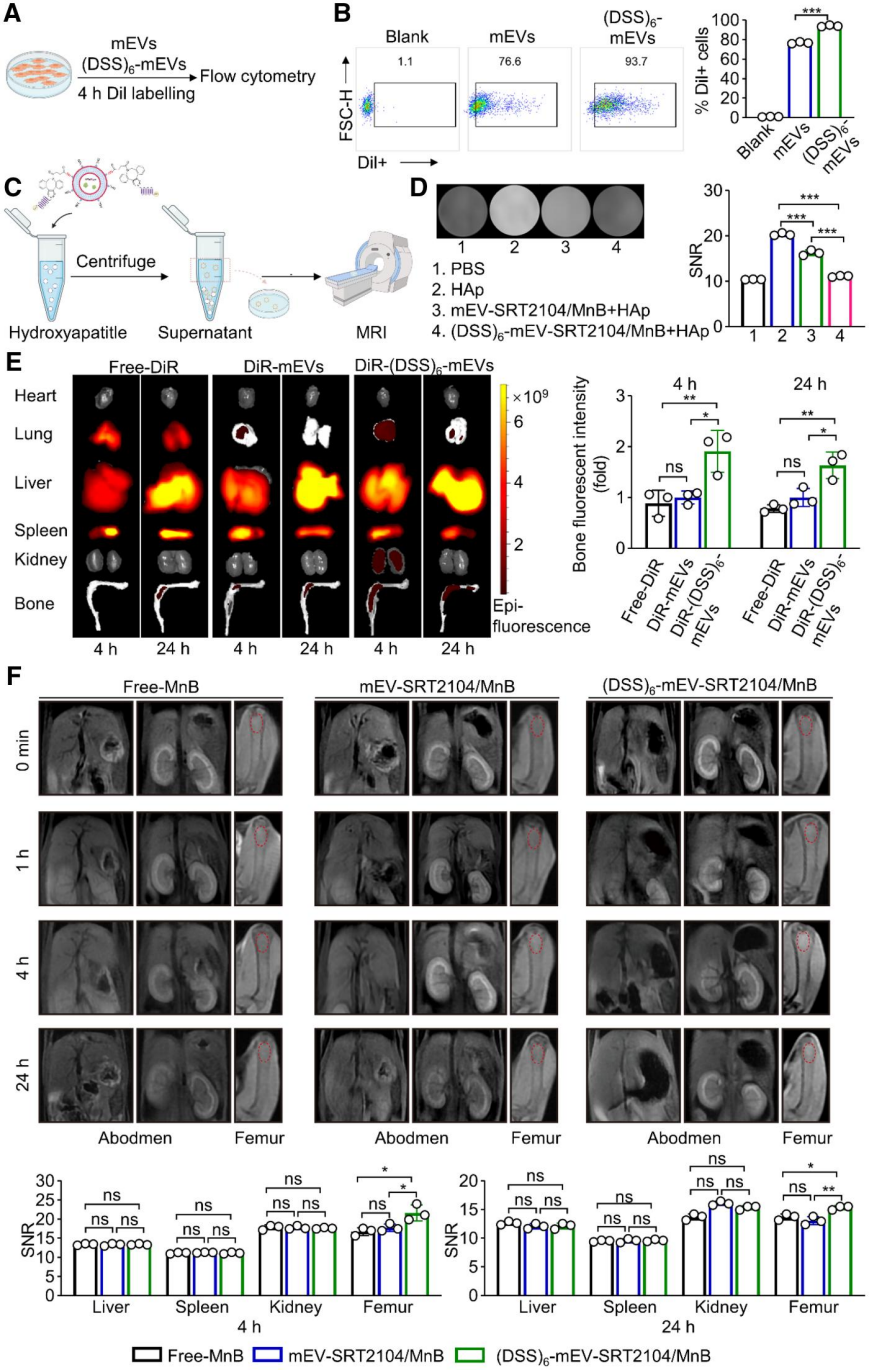
Bone-targeting capability of engineered mEVs *in vitro* and *in vivo*. (**A**) Schematic illustration of the culture of MC3T3-E1 cells. (**B**) Uptake of Dil-labelled (DSS)_6_-mEVs with MCST3-E1 cells by flow cytometry. (**C**) Schematic illustration of the binding of (DSS)_6_-mEV-SRT2104/MnB and mEV-SRT2104/MnB with hydroxyapatite. (**D**) T_1_-weighted MR images of the supernatant after the binding of mEVs or (DSS)_6_-mEVs with hydroxyapatite. (**E**) *In vivo* biodistribution of DiR-labelled (DSS)_6_-mEVs or mEVs by IVIS. (**F**) *In vivo* biodistribution of mEV-SRT2104/MnB or (DSS)_6_-mEV-SRT2104/MnB by MRI (ns, no significant; *P < 0.05, **P < 0.01, ***P < 0.001).

Biomaterials have been successfully used in the therapy of osteoporosis, but their bone-targeting abilities have hindered their further development. A bone-targeted therapy aims to achieve high drug concentrations at the lesion site to produce better treatment results without causing obvious side effects [42]. To evaluate the bone-targeting capability of (DSS)_6_, Dil-labelled (DSS)_6_-mEVs or mEVs were incubated for 4 h (Figure 3A). As illustrated in Figure 3B, the percentage of Dil+ cells in (DSS)_6_-mEVs-treated osteoblasts was higher than mEVs (93.7% vs. 76.6%), demonstrating an osteoblast-specific uptake of (DSS)_6_-mEVs. We further confirmed this result using a fluorescence microscope and the result is shown in Supplementary Figure S2. It was found that MC3T3-E1 cells could uptake more (DSS)_6_-mEVs than mEVs.

To identify the ability of engineered mEVs to bind with low-crystallinity HAp, (DSS)_6_-mEV-SRT2104/MnB and mEV-SRT2104/MnB were incubated with HAp suspensions, followed by centrifugation to separate the unbound HAp (Figure 3C). The supernatants were collected and imaged using a 3.0 T clinical MRI scanner. In comparison with mEV-SRT2104/MnB, the T_1_ imaging of (DSS)_6_-mEV-SRT2104/MnB became darker following the removal of bound mEVs. Quantitative analysis of the MR SNR showed that the SNR of the (DSS)_6_-mEV-SRT2104/MnB decreased from 20.5 (pre-incubated) to 11.1 at post-incubated, while the SNR of the mEV-SRT2104/MnB decreased from 20.3 to 16.2 during the same incubation period (Figure 3D).

The *in vivo* distribution of (DSS)_6_-mEV was investigated with IVIS. DiR-labelled (DSS)_6_-mEVs and mEVs were intravenously injected into mice with an equal quantity of free-DiR used as control. Then the major organs, tibia and femur were harvested for imaging after 4 and 24 h. As displayed in Figure 3E, the bone of (DSS)_6_-mEVs-treated mice showed a stronger fluorescent signal compared to mEVs-treated and free-DiR-treated mice. A quantitative analysis was performed on fluorescence signal intensity, it showed the fluorescent intensity in (DSS)_6_-mEVs-treated mice upregulated by 1.9 folds and 1.6 folds for 4 and 24 h compared to mEVs-treated mice, respectively. MRI was then used to visualize the distribution of free MnB NPs, mEV-SRT2104/MnB and (DSS)_6_-mEV-SRT2104/MnB *in vivo*, which would disrupt the relaxation of nearby water protons, decreasing T1 relaxation times and T1 signal intensity (Figure 3F and Supplementary Figure S3). The MRI results for the liver, spleen and kidney indicated a rise in signal enhancement after 30 min injection, which could be corresponded to the accumulation of MnB NPs or engineered mEVs in the organ, followed by a gradual decrease. There were no significant differences in abdominal MR signal intensities at different time points among the three groups. The T_1_-weighted MR contrast changes in the femur were not prominent in both the free MnB NPs and mEV-SRT2104/MnB groups. However, the MR signal intensity of the distal femur was enhanced after 4 h of (DSS)_6_-mEV-SRT2104/MnB injection, with this enhancement persisting for over 24 h which was in accord with the results of fluorescence imaging. The results revealed that modification with (DSS)_6_ could effectively facilitate bone accumulation of engineered mEVs.

### OVX mouse model establishment

**Figure 4.**
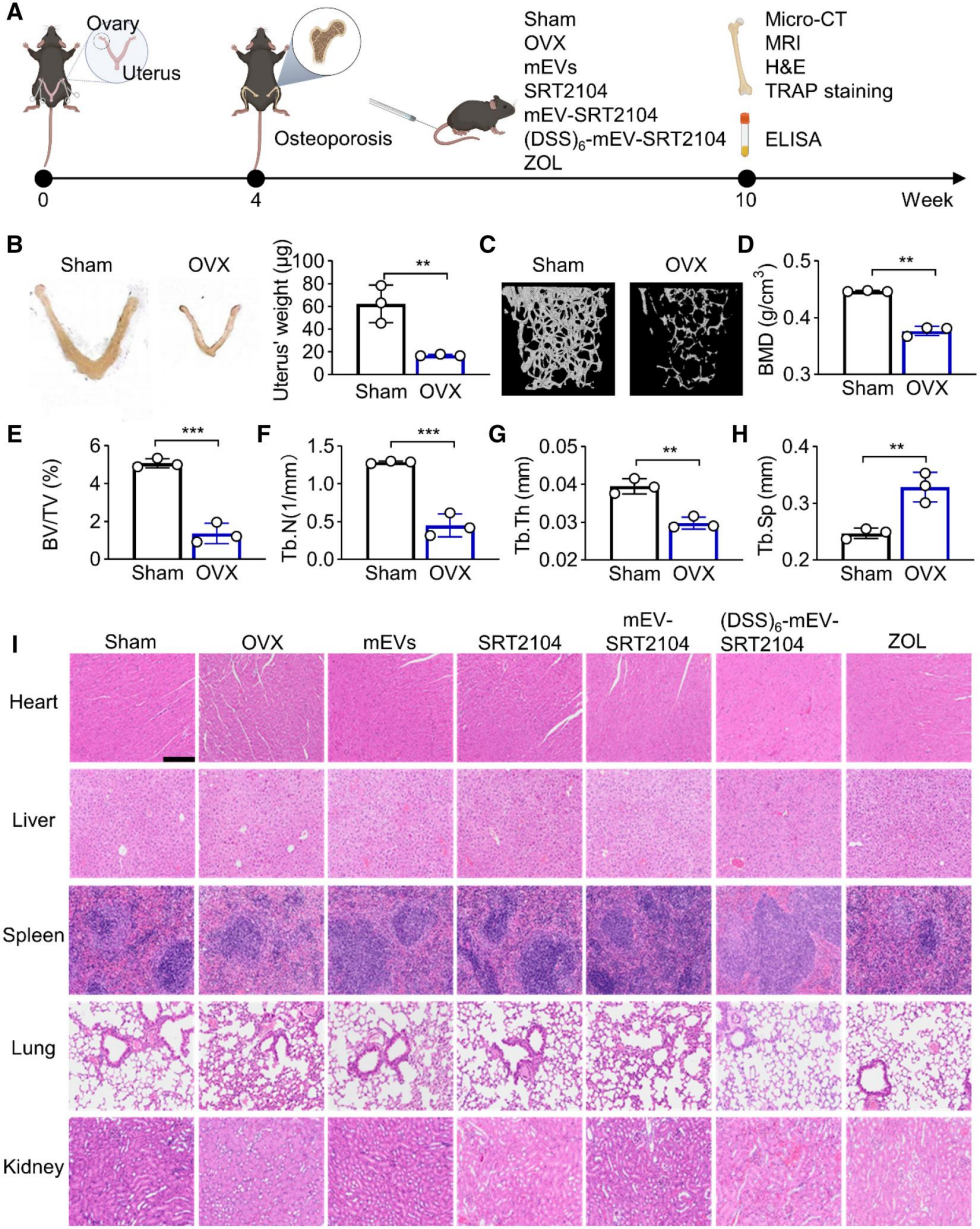
Establishment of OVX mouse model and *in vivo* biocompatibility evaluation. (**A**) The schematic illustration of the *in vivo* studies. (**B**) Representative uterus images of mice 4 weeks after surgery and quantitative results of uterus’ weight. (**C**) Representative micro-CT images showing 3D microarchitecture of trabeculae in the distal femurs. (**D–H**) Characterizations of the BMD, BV/TV, Tb.N, Tb.Th and Tb.Sp of distal femurs 4 weeks after surgery. (**I**) H&E staining of the main organs in different groups after 6 weeks of treatment to OVX mice. Scale bar = 250 μm (**P＜0.01, ***P＜0.001).

First, OVX mouse models were established to simulate bone loss caused by estrogen deficiency. After four weeks, a substantial reduction in uterine size and weight was observed in the OVX group, with a 73% decline compared to sham controls (Figure 4B). This was attributed to the sudden drop in estrogen levels following ovarian removal. Micro-CT analysis of the femur (Figure 4C) revealed significant bone loss in the OVX group, characterized by lower BMD (0.38 ± 0.007 vs. 0.45 ± 0.001 g/cm^3^), BV/TV (1.36 ± 0.44 vs. 5.08 ± 0.20%) and Tb.N (0.45 ± 0.12 vs. 1.29 ± 0.01 mm^−1^) (Figure 4D–H). The OVX model was successfully established based on these results.

### *In vivo* biocompatibility evaluation of (DSS)_6_-mEV-SRT2104

We next evaluated the *in vivo* biocompatibility of mEVs, SRT2104, mEV-SRT2104 and (DSS)_6_-mEV-SRT2104, which were intravenously injected twice a week for 6 weeks to the established OVX mice. ZOL was intravenously injected once according to the user’s guide. As shown in Figure 4I, the organs did not exhibit apparent histomorphometric changes, indicating that the treatment was biocompatible. Moreover, we recorded the body weight of the mice during the treatment (Supplementary Figure S4). There was no significant difference in mouse body weight between the OVX group and the treated groups, indicating no obvious toxicity occurred in all the groups.

### (DSS)_6_-mEV-SRT2104 reduces bone loss in OVX mice by dual regulation of bone remodeling

**Figure 5.**
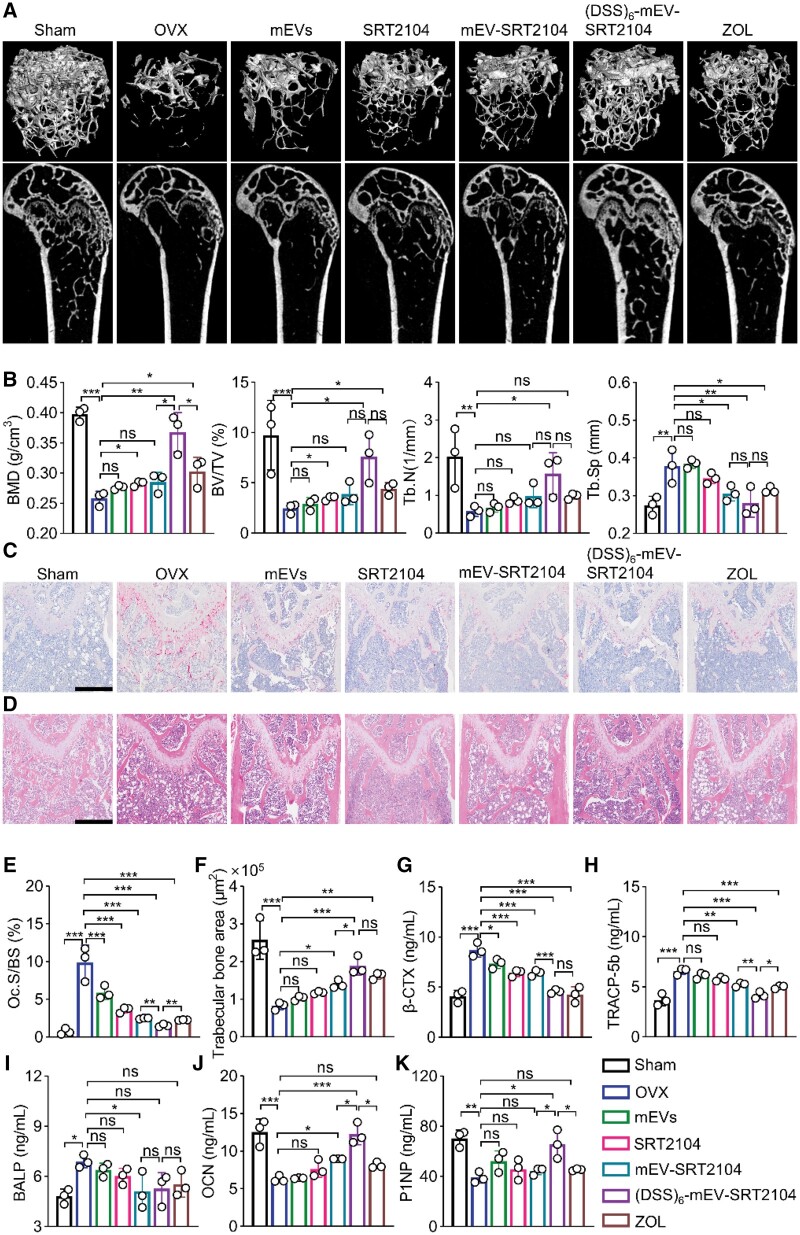
(DSS)_6_-mEV-SRT2104 reduces bone loss in OVX mice by dual regulation of bone remodeling. (**A**) Representative micro-CT images showing 3D microarchitectures of trabeculae in the distal femurs. (**B**) Micro-CT quantitative analysis of BMD, BV/TV, Tb.N and Tb.Sp of distal femurs. (**C**) TRAP staining of bone sections. Scale bar = 250 μm. (**D**) H&E staining of bone sections of experimental mice. Scale bar = 250 μm. (**E**) Histomorphometric analysis of osteoclast surface per bone surface (Oc.S/BS). (**F**) Histomorphometric analysis of the trabecular bone area. (**G–K**) The relative serum protein expression evaluated by the Elisa test, including β-CTX (**G**), TRACP-5b (**H**), BALP (**I**), OCN (**J**) and P1NP (**K**) (ns, no significance; *P < 0.05, **P < 0.01, ***P < 0.001).

Next, the *in vivo* therapeutic efficacy of (DSS)_6_-mEV-SRT2104 was further evaluated as well. The 3D reconstructed images and coronal sections revealed significant bone loss in OVX mice, contrasting with denser femoral metaphyseal structure in other groups, especially those administrated with (DSS)_6_-mEV-SRT2104 (Figure 5A). Quantitative bone morphometric parameters confirmed the superior performance of (DSS)_6_-mEV-SRT2104, exhibiting significantly higher BMD (0.37 ± 0.03 g/cm^3^ vs. 0.26 ± 0.01 g/cm^3^), BV/TV (7.59 ± 1.98% vs. 2.48 ± 0.42%), Tb.N (1.58 ± 0.45 mm^−1^ vs. 0.58 ± 0.12 mm^−1^) and lower Tb.Sp (0.28 ± 0.03 mm vs. 0.38 ± 0.04 mm) compared to the OVX group (Figure 5B). Comparable results were observed in the ZOL group, yet (DSS)_6_-mEV-SRT2104 exhibited significantly higher BMD (0.37 ± 0.03 g/cm^3^ vs. 0.30 ± 0.02 g/cm^3^). These findings strongly indicated that (DSS)_6_-mEV-SRT2104 can effectively enhance bone density and promote bone regeneration.

To assess the *in vivo* effect of (DSS)_6_-mEV-SRT2104 on osteoclast activity, TRAP staining was performed. Significant reductions in excessive osteoclastogenesis (Oc.S/BS) were found in the results, with 1.53 ± 0.16% in the (DSS)_6_-mEV-SRT2104 group compared to 9.87 ± 1.86% in the OVX group (Figure 5C and E), indicating that (DSS)_6_-mEV-SRT2104 treatment inhibited osteoclastogenesis, thereby preventing excessive bone loss to enhance osseointegration. This was further corroborated by H&E staining and micro-CT analysis, revealing substantial trabecular bone loss in the OVX group compared to the sham and treatment groups (Figure 5D and F). Notably, (DSS)_6_-mEV-SRT2104 treatment significantly increased trabecular bone area (188405.30 ± 19633.40 μm^2^ vs. 82232.51 ± 8224.03 μm^2^) in the OVX group, suggesting its great potential in preventing OVX-induced osteoporosis.

To further evaluate the mechanism underlying the bone restoration induced by (DSS)_6_-mEV-SRT2104, we analysed serum markers specific bone resorption (β-CTX, TRACP-5b) and bone formation (BALP, OCN and P1NP) (Figure 5G–K). In OVX mice, TRACP-5b and BALP activities had a significant increase, which indicates an increase in osteoclast activity and remodeling of bone tissue. After (DSS)_6_-mEV-SRT2104 treatment, β-CTX and TRACP-5b were reduced, while OCN and P1NP increased, approaching normal levels. It was found that (DSS)_6_-mEV-SRT2104 inhibited bone resorption and stimulated bone synthesis, thereby preventing bone loss.

### The T_1_-weighted MRI of (DSS)_6_-mEV-SRT2104/MnB in OVX mice

**Figure 6.**
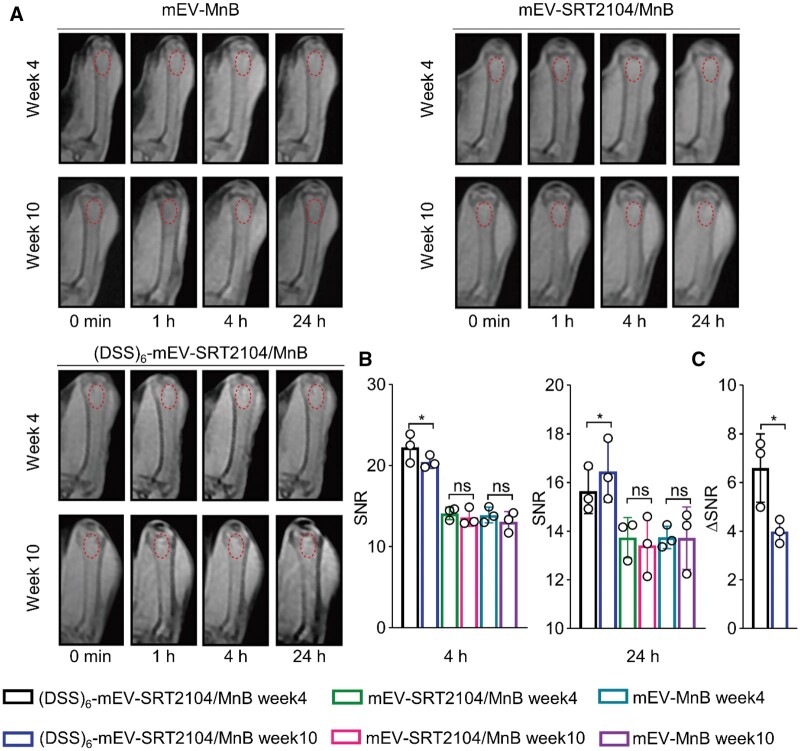
T_1_-weighted MRI of OVX mice. (**A**) The femur T_1_-weighted MR images were acquired from OVX mice administered with mEV-MnB, mEV-SRT2104/MnB or (DSS)_6_-mEV-SRT2104/MnB at various time points during week 4 and 10. (**B**) SNR analysis of (a). (**C**) The ΔSNR from 4 to 24 h in the distal femur of OVX mice administered with (DSS)_6_-mEV-SRT2104/MnB was assessed based on T_1_-weighted MRI images (ns, no significance; *P ＜ 0.05).

We then investigated the bone-targeting ability of (DSS)_6_-mEV-SRT2104/MnB in OVX mice using MRI. The OVX mice were administered with mEV-MnB, mEV-SRT2104/MnB or (DSS)_6_-mEV-SRT2104/MnB *via* tail vein injection at various time points during week 4 and 10 following OVX surgery. T_1_-weighted MRI examination revealed significant accumulation of (DSS)_6_-mEV-SRT2104/MnB in the distal femur at both week 4 and 10 (Figure 6A, and Supplementary Figure S5). The femur SNR analysis showed a substantial increase in signal enhancement for (DSS)_6_-mEV-SRT2104/MnB 4 h post-injection compared to the mEV-MnB or mEV-SRT2104/MnB groups. This finding unequivocally validated the superior bone-targeting efficacy of (DSS)_6_-mEV-SRT2104/MnB in OVX mice (Figure 6B).

Subsequently, we quantified the signal intensity dynamics of (DSS)_6_-mEV-SRT2104/MnB in OVX mice by measuring the change in 4–24 h SNR (ΔSNR) in the distal femur based on T_1_-weighted MRI images. Notably, the ΔSNR at week 4 (7.24 ± 1.41) exhibited a statistically significant elevation compared to the value at week 10 (3.99 ± 0.49) (Figure 6C). Collectively, the above results underscored the dynamic behavior of (DSS)_6_-mEV-SRT2104/MnB in OVX mice, indicating a more rapid *in vivo* change at the beginning of treatment compared to the later stages of therapy.

## Discussion

In clinical practice, several widely used pharmacological agents effectively mitigated the risk of osteoporosis by primarily reducing bone resorption, yet prolonged use may increase the risk of side effects. To address the challenges of imbalanced bone homeostasis and the limitations of anti-resorptive drugs in osteoporosis treatment, there is a pressing need to develop a strategy targeting both bone formation and resorption [43].

In our study, SRT2104 significantly promoted MC3T3-E1 cell osteogenesis as evidenced by ALP staining and inhibited the formation of TRAP-positive multinucleate cells *in vitro* (Figure 2), indicating its potential for osteoporosis treatment. However, *in vivo* administration of SRT2104 did not significantly reverse OVX-induced bone loss (Figure 5). This may be attributed to the inherent properties of bone tissue, such as its relative inflexibility, low permeability and blood circulation, which limited the application of SRT2104 [44, 45].

To enhance the therapeutic efficacy of SRT2104 *in vivo*, we developed engineered mEVs by modifying them with a bone-targeting peptide. mEVs were known to enhance bone repair and promote the expression of osteogenic genes [28, 46]. Additionally, among various EVs, mEVs are abundant in nature and exhibit many characteristics favorable for theranostic applications, such as surface engineering for organ- or tissue-specific delivery [47]. Taking the above into account, we then conjugated (DSS)_6_ to the surface of mEVs *via* click chemistry. In contrast to previously reported bone-targeted moieties, (DSS)_6_ peptide demonstrated higher bone-specific targeting efficiency and improved biosafety [48, 49]. Furthermore, compared to non-covalent modification methods, click chemistry offers a more stable and efficient modification approach [34, 50]. Our study revealed that bone-targeted mEVs accumulated significantly at the distal femur, confirmed by bioluminescence imaging and MRI, possibly due to the abundant blood supply in the trabecular bone at the metaphysis and its relatively fast bone turnover rate [45, 51]. Micro-CT analysis of the femur revealed that the bone-targeted mEVs group achieved the highest BV/TV ratio. Additionally, histological analysis showed that the bone-targeted mEVs group possessed the lowest OC.S/BS and largest trabecular bone area. ELISA results further indicated that the bone-targeted mEVs group augmented the expression of bone formation markers while inhibiting bone resorption markers (Figure 5). Meanwhile, with the loading of MnB NPs into mEVs, (DSS)_6_-mEV-SRT2104/MnB could serve as targeted nanotheranostics for simultaneous therapy and MRI. Furthermore, the *in vivo* behavior of (DSS)_6_-mEV-SRT2104/MnB under MRI monitoring may potentially reflect the therapeutic effect. As compared to week 4, week 10’s femur ΔSNR was significantly lower (Figure 6). The improvement in imbalanced bone homeostasis post-treatment led to a reduced clearance rate of engineered mEVs-attached hydroxyapatite [51]. In summary, we believe the reported engineered mEVs, with their enhanced bone-targeting capability and theranostic efficacy, presented as a promising strategy for osteoporosis therapy and monitoring.

## Conclusion

In this study, engineered mEVs (DSS)_6_-mEV-SRT2104/MnB was constructed *via* surface modification of bone-targeting peptide and loading with SRT2104 and MRI contrast agent MnB NPs for the therapy and monitoring of osteoporosis. Physicochemical characterizations of the engineered mEVs confirmed the successful purification and modification of the engineered mEVs. Furthermore, both *in vitro* and *in vivo* studies demonstrated superior performance in bone restoration by inhibiting bone resorption and stimulating bone formation. Meanwhile, the loading of MRI contrast agent offers visualized monitoring during the treatment. Overall, we believe (DSS)_6_-mEV-SRT2104/MnB provides a new paradigm for the therapy and monitoring of osteoporosis.

## Supplementary Material

rbae112_Supplementary_Data
