# Supplementary material for: Bone-targeting engineered milk-derived extracellular vesicles for MRI-assisted therapy of osteoporosis
Source: Regen Biomater. 2024 Sep 13;11:rbae112. doi: 10.1093/rb/rbae112 (PMC11422186; doi:10.1093/rb/rbae112)
Supplement: rbae112_Supplementary_Data [file rbae112_supplementary_data.docx]

**Supplementary Information**

**Bone-targeting Engineered Milk-derived Extracellular Vesicles for MRI-assisted Therapy of Osteoporosis**

Qing Huang^1,3 †^, Yang Jiang^2, 3†^, Yang Cao^1, †^, Yunchuan Ding^1^, Jinghui Cai^2^, Tingqian Yang^2^, Xin Zhou^3^, Qiang Wu^3^, Danyang Li^3,^ *, Qingyu Liu^2,^ *, Fangping Li^1,^ *

^1^ Department of Endocrinology, The Seventh Affiliated Hospital, Sun Yat-sen University, Shenzhen 518107, China

^2^ Department of Radiology, The Seventh Affiliated Hospital, Sun Yat-sen University, Shenzhen 518107, China

^3^ Research Center, The Seventh Affiliated Hospital, Sun Yat-sen University, Shenzhen 518107, China

* Correspondence address.

Email:

lidy55@mail.sysu.edu.cn (D.L.)

liuqingyu@sysush.com (Q.L.)

lifangping@sysush.com (F.L.)

^†^ These authors contribute equally.

**
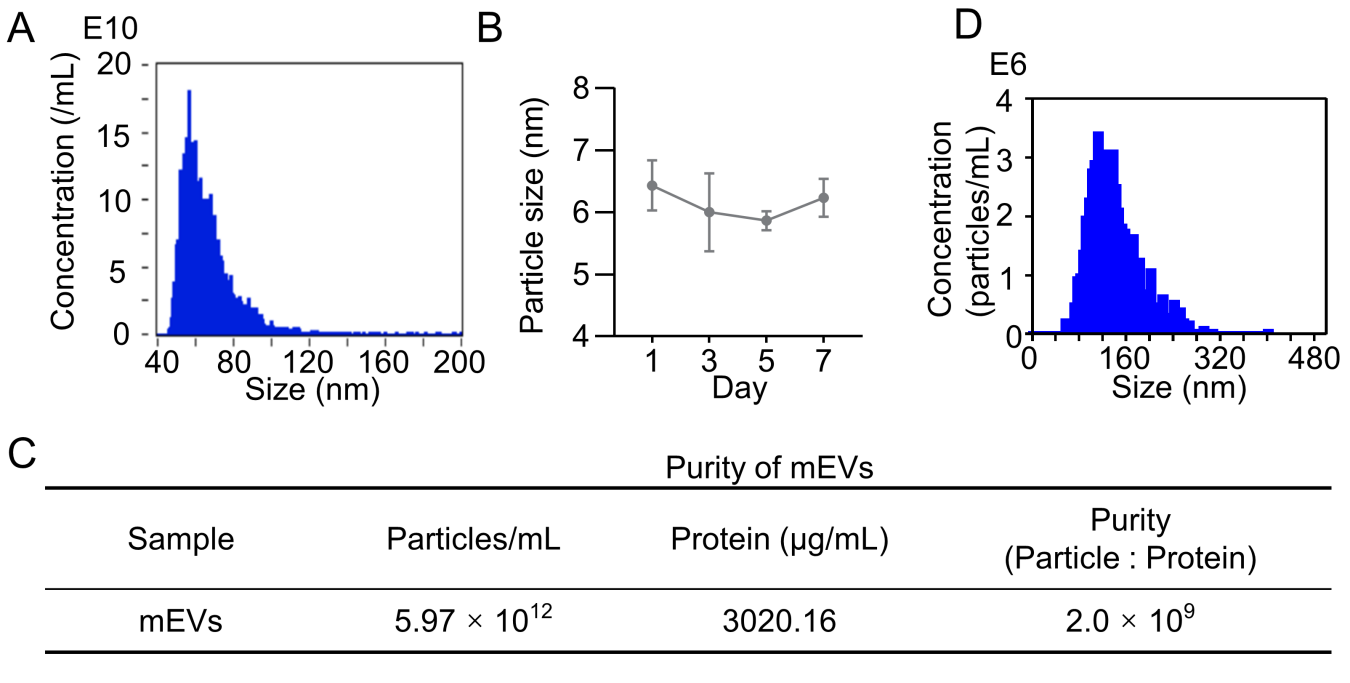
**

**Figure S1.** Characterization of mEVs and MnB NPs. **(A)** Size of mEVs measrured with nanoflow. **(B)** Hydrodynamic size of MnB NPs for stability assessment during 7 days. **(C)** Purity calculation of the obtained mEVs. **(D)** Size of (DSS)_6_-mEV-SRT2104/MnB measured with nanoflow.


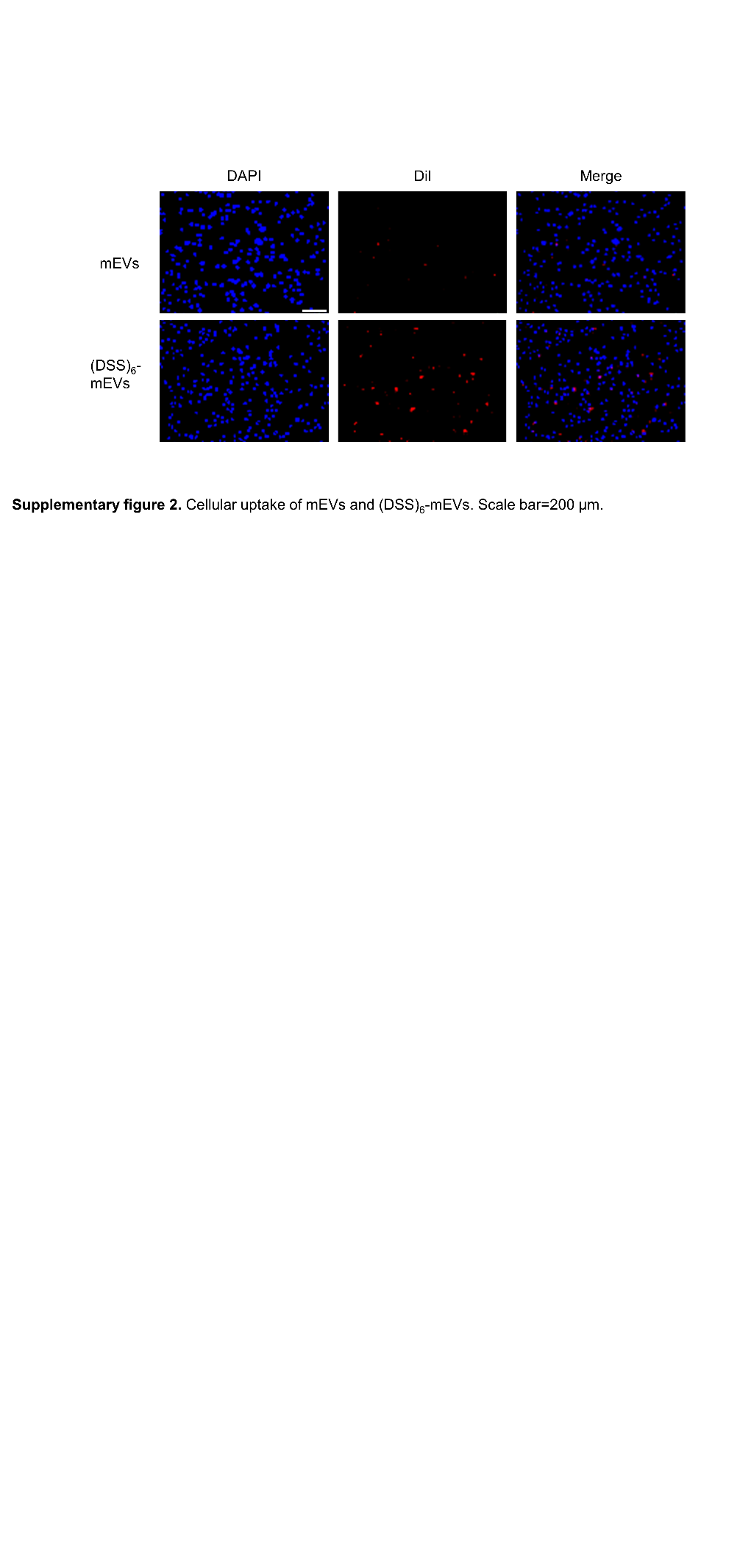
 **Figure S2.** Images of the cellular uptake of mEVs and (DSS)_6_-mEVs. Scale bar=200 μm.


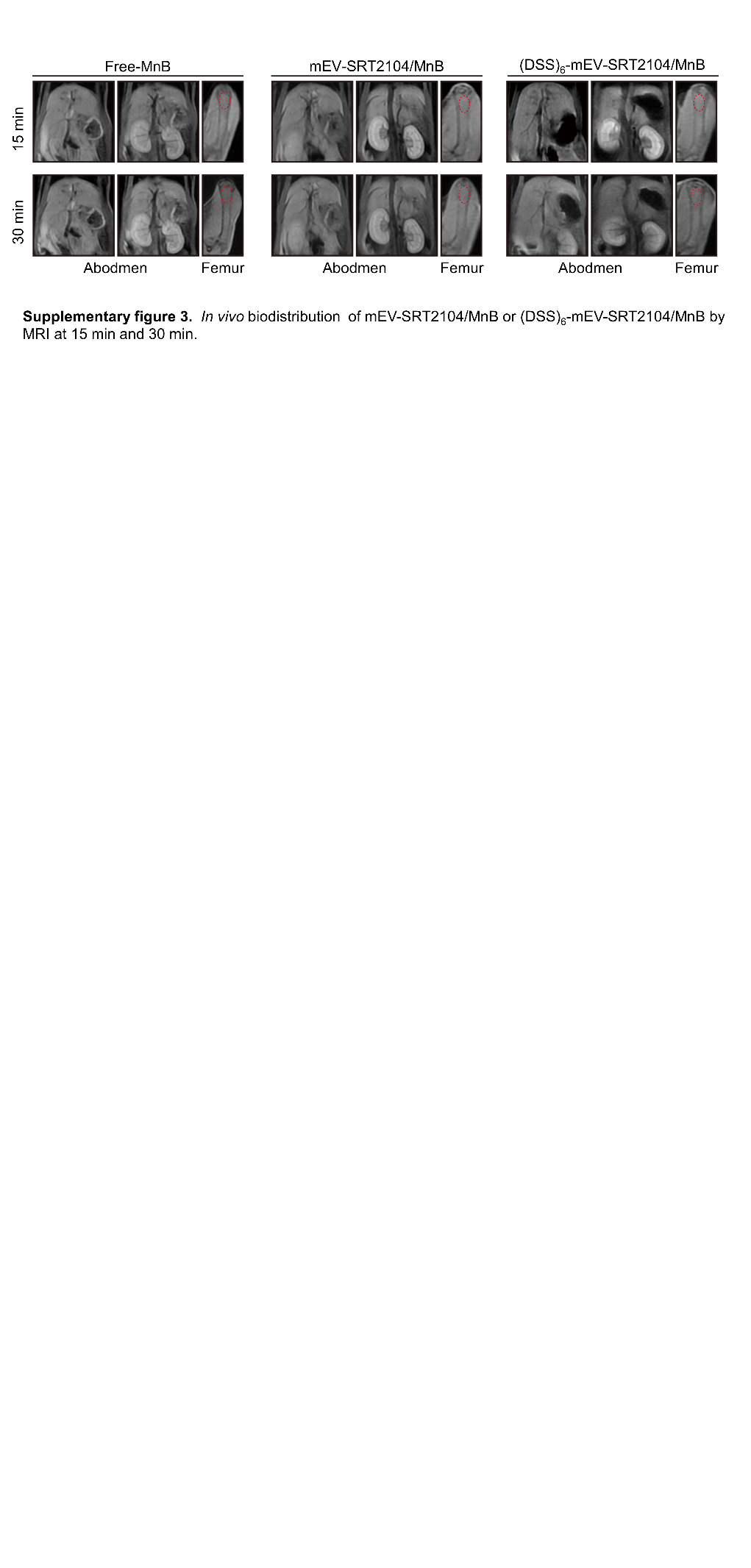


**Figure S3.** *In vivo* biodistribution of mEV-SRT2104/MnB or (DSS)_6_-mEV-SRT2104/MnB by MRI.


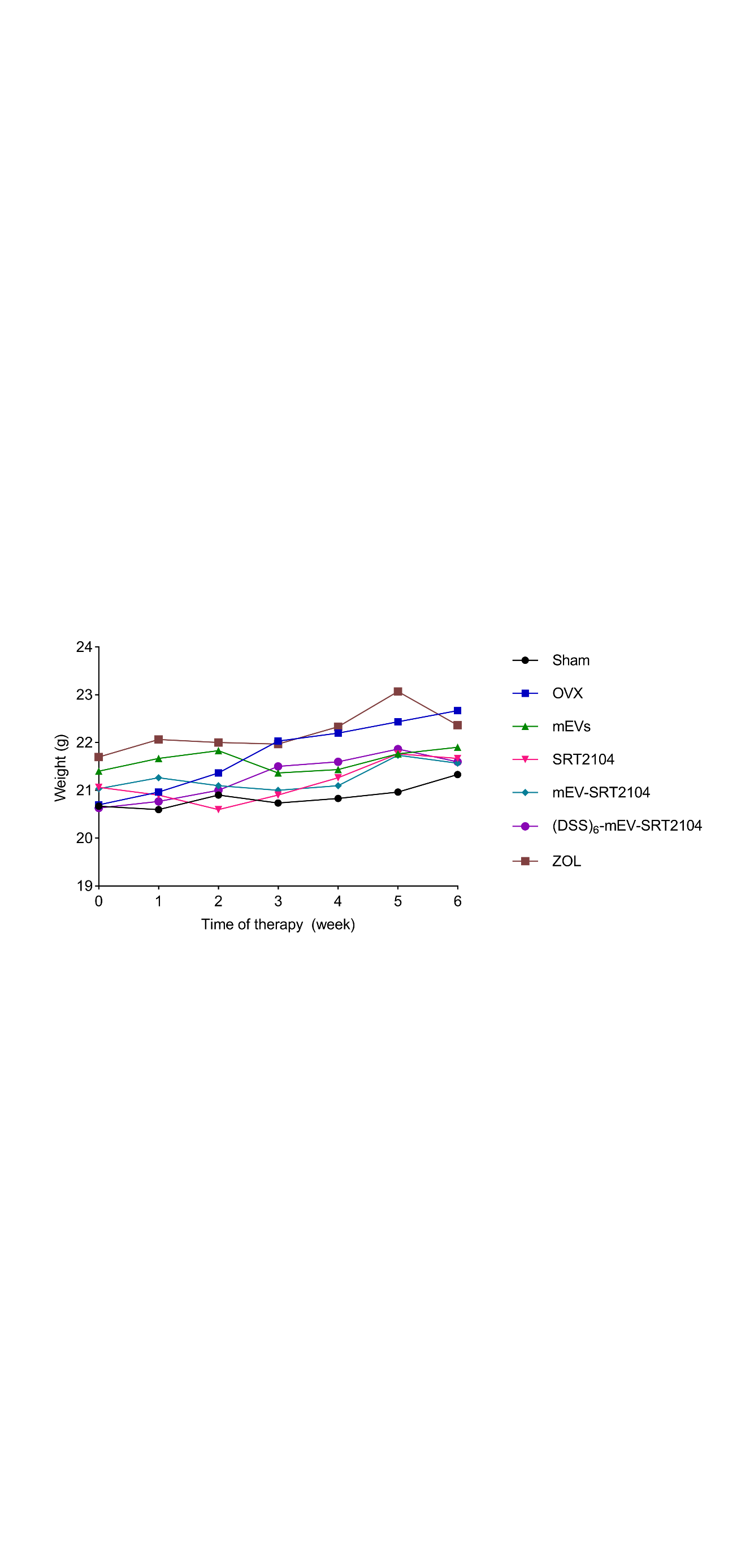


**Figure S4.** The body weight of the mice.


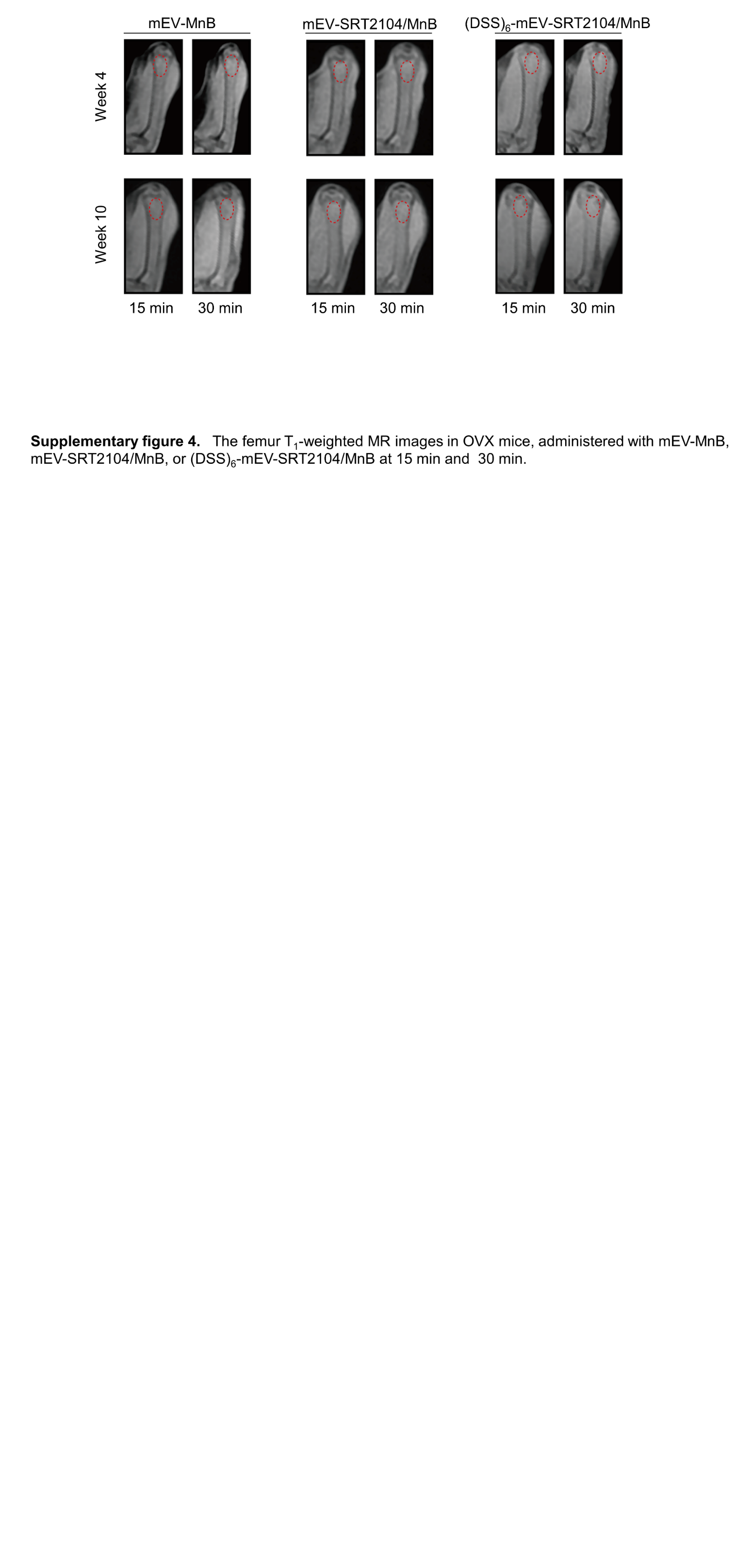


**Figure S5.** The femur T_1_-weighted MR images in OVX mice, administered with mEV-MnB, mEV-SRT2104/MnB, or (DSS)_6_-mEV-SRT2104/MnB.
